# Confocal laser endomicroscopy to monitor the colonic mucosa of mice

**DOI:** 10.1016/j.jim.2015.04.012

**Published:** 2015-06

**Authors:** Lisa Mielke, Adele Preaudet, Gabrielle Belz, Tracy Putoczki

**Affiliations:** a The Walter and Eliza Hall Institute for Medical Research, Melbourne, Australia; b The Department of Medical Biology, University of Melbourne, Australia

**Keywords:** MEICS, Murine Endoscopic Index of Colitis Severity, Colon, Endoscopy, Epithelium, Inflammation, Mouse models, Vasculature

## Abstract

The gastrointestinal tract is a unique organ system that provides an epithelial barrier between our underlying immune system and luminal pathogens. Disruption of gastrointestinal homeostasis, as a result of impaired barrier function, is associated with numerous pathologies including inflammatory bowel disease and colorectal cancer. In parallel to the clinical development of endoscopy technologies to monitor and diagnose these pathologies in humans, advanced mouse colonoscopy techniques are being developed. When these technologies are coupled with model systems of human disease, which are essential to our understanding of the pathophysiology of gastrointestinal diseases, the requirement for euthanasia of multiple cohorts of mice is eliminated. Here we highlight the suitability of white light endoscopy to monitor the progression of colitis in mice. We further outline the experimental power of combined standard endoscopy with confocal microendoscopy, which permits visualization of fluorescent markers in a single animal in real-time. Together, these technologies will enhance our understanding of the interplay between components of the gastrointestinal microenvironment and their role in disease.

## Highlights

• Monitoring of mucosal damage using white light endoscopy
• Monitoring of the epithelial barrier using confocal endomicroscopy
• Monitoring of the vasculature architecture using confocal endomicroscopy

## 1 Introduction

The gastrointestinal tract forms an essential barrier that protects the body from invasive microorganisms while performing the intricate task of digesting and absorbing nutrients from the food we consume. Crosstalk between intestinal epithelial cells (IEC) and the immune system plays a critical role in maintaining barrier function by detecting and responding to environmental stimuli including pathogens, dietary metabolites, food antigens and the commensal bacteria that comprise the microbiota. Breakdown of these networks leads to intestinal immunopathology, including infection, coeliac disease, and inflammatory bowel disease (IBD).

IBD describes a group of relapsing–remitting inflammatory disorders, including Crohn's disease (CD) and ulcerative colitis (UC). As a result of chronic bowel inflammation, IBD patients have an increased risk of developing colorectal cancer (CRC) depending on genetic and environmental factors as well as the severity and duration of the disease. CRC is one of the leading causes of cancer-related deaths worldwide; however, if colon polyps are detected early using techniques such as colonoscopy, the removal of these pre-cancerous lesions can dramatically reduce the incidence of advanced CRC (Brenner et al., 2014). While significant progress has been made towards identifying colon polyps by endoscopy, dysplasia or flat polyps that predispose to malignant disease are often difficult to discriminate by standard colonoscopy. Therefore continued development of powerful imaging techniques will be the key to increased detection of subtle changes in the mucosa, which will enhance the diagnosis and treatment of intestinal immunopathologies, including CRC.

Recent innovative techniques combining traditional white light endoscopy with fluorescence endoscopy have enhanced our understanding of the pathogenesis of IBD and increased our ability to detect dysplasia and precancerous lesions of the gastrointestinal tract (Atreya et al., 2014, Coda and Thillainayagam, 2014). These new techniques involve imaging of the colonic mucosa following topical administration of fluorescein or fluorescently-conjugated antibodies to permit visualization of specific subsets of immune cell populations, endothelial or epithelial cells. The introduction of fluorescent capabilities provides both clinicians and basic scientists with the opportunity to track the recruitment of cell populations to an ulcer for example, in real-time, as opposed to histopathological analysis of biopsies.

Standard white light endoscopy is also increasingly used as an experimental tool with genetic or chemically induced mouse models of IBD and CRC to grade inflammation and monitor tumor development in an individual animal over time. These mouse models mimic different features of the pathophysiology of intestinal diseases, including loss of epithelial barrier integrity, chronic inflammation and epithelial hyperplasia. Here we describe new confocal laser endomicroscopy methods suitable for use in mice, which take advantage of the combined used of standard white light endoscopy and confocal microendoscopy. These novel methods permit the generation of high-magnification cross-sectional images of the colonic mucosa for immediate visualization of immune, endothelial or epithelial cell behavior without the need for a biopsy or euthanasia and tissue collection.

## 2 Materials and methods

### 2.1 Equipment

2.1.1 Karl Storz Coloview miniendoscopic system
  2.1.1.1 Endovision Tricam and 3-Chip camera head (cat # 20223011, 20221030)
  2.1.1.2 Xenon 175 light source with anti-fog pump (cat # 20131501)
  2.1.1.3 Hopkins straight forward telescope, diameter 1.9 mm (cat # 64301A)
  2.1.1.4 Examination and protection sheath (cat # 61209C)
  2.1.1.5 Fiber optic light cable and air hose (cat # 495NL)
  2.1.1.6 Computer and standard video software
2.1.2 CellVizio Dual Band confocal endoscopy system
  2.1.2.1 Confocal processor (cat# CDB-0001)
  2.1.2.2 Proflex microprobe, Mini-Z, diameter 0.94 mm
2.1.3 Isoflurane machine
  2.1.3.1 Induction box
  2.1.3.2 Nose cone

### 2.2 Materials

2.2.1 Dextran sulfate sodium (DSS; mol wt. 36,000–50,000)
2.2.2 FIuorescein isothiocyanate–dextran (FITC–dextran; average mot wt. 70,000; Sigma)

### 2.3 Mice and housing

2.3.1 All procedures were approved and conducted in accordance with the Walter and Eliza Hall Institute Animal Ethics Committee.
2.3.2 To minimize variation in gut microflora, all animals within an experimental cohort should be bred in the same room and housed on the same rack in a specific pathogen-free barrier facility.

### 2.4 Induction of mucosal damage

2.4.1 Baseline weights should be determined for individual mice on Day 0.
2.4.2 Prepare 2% (w/v) DSS in sterile mouse drinking water, allowing for approximately 5 mL of water per mouse/day of the experiment.
2.4.3 Provide DSS water to the mice for 5 consecutive days.
2.4.4 On Day 5 return the mice to normal drinking water for a further three days.
2.4.5 Throughout the 8 day cycle, monitor mice for weight-loss, stool consistency, the presence of blood in the stools, lethargy, ruffled fur, hunching and reduced movement.
2.4.6 Animals that experience > 15% transient weight-loss over three days should be ethically euthanized.

### 2.5 Monitoring the mucosal barrier by serial white-light endoscopy

2.5.1 The Coloview white light endoscopy equipment and the CellVizio confocal endoscopy equipment should be assembled according to the manufacturer's guidelines.
2.5.2 Clean and sterilize the Karl Storz endoscopy sheath using 70% ethanol and an anti-bacterial agent.
2.5.3 Anesthetize live mice in an induction chamber with 3% isoflurane and 100% oxygen at a flow rate of 0.4 L/min.
2.5.4 Secure the head of the mouse in a nose cone, with the mouse positioned ventral side up and the hind legs comfortably stretched out behind.
2.5.5 Maintain the isoflurane at 0.5–2% for the endoscopy procedure.
2.5.6 Confirm the rate of airflow by placing the tip of the endoscopy sheath in a beaker of water. One air bubble at a time should emerge from the sheath.
2.5.7 Insert the endoscope sheath up to 3 cm into the rectum.
2.5.8 Record endoscopy videos using standard media software, such as iMovies.
2.5.9 Record clinical disease scores for the Murine Endoscopic Index of Colitis Severity (MEICS). Scoring parameters are provided in Fig. 2.

### 2.6 Monitoring the mucosal barrier by confocal endoscopy

2.6.1 Initialize the CellVizio 488 laser scanning unit, turn on the confocal processor and launch the CellVizio Dual and ImageCell software. The laser requires approximately 20 min to initialize.
2.6.2 Insert a microprobe of choice into the laser scanning unit. Use the Quantikit 488 to calibrate the probe as per the manufacturer instructions, and adjust the laser power to approximately 30%.
2.6.3 Insert the fiber optic probe down the working channel of the Karl Storz endoscopy sheath.
2.6.4 Initiate video recording from the Karl Storz Coloview system, and insert the endoscopy sheath up to 3 cm into the rectum of the mouse. Place the sheath against the colonic mucosa for fluorescent imaging.
2.6.5 Initiate the 488 laser using the foot pedal and use the ImageCell software to record fluorescent videos in real-time. Simultaneous recording using the white light endoscopy unit is also possible.
2.6.6 Re-calibrate the system following the manufacturer protocols prior to imaging each mouse.
2.6.8 Place the mouse back in its cage and monitor for recovery from anesthetic.

### 2.7 Monitoring the mucosal vasculature by confocal endoscopy

2.7.1 Prior to fluorescent imaging, inject 100 μL FITC–dextran (5% w/v; Sigma) intravenously.
2.7.2 Monitor the time after injection, image the colon as described in Section 2.6, and record the time of imaging.
2.7.3 CellVizio Vessel Detection software can be used to quantify vessel segmentation, length, area and diameter.

### 2.8 Cleaning of the endoscopy units

2.8.1 Clean the Karl Storz endoscopy sheath, Karl Storz light probe and CellVizio fiber optic probes thoroughly in accordance with manufacturer suggestions.
2.8.2 Disassemble and store the equipment as per the manufacturer suggestions.

## 3 Theory

The increased use of mouse colonoscopy over the past decade has allowed us to monitor clinical disease progression in an individual mouse over time. The recent development of new imaging technologies, including miniaturized confocal laser endomicroscopy, has enabled high resolution in vivo tracking of fluorescently labeled proteins throughout the course of disease. These advancements will allow for an enhanced understanding of the pathophysiology of IBD and CRC.

## 4 Results

**Fig. 1:**
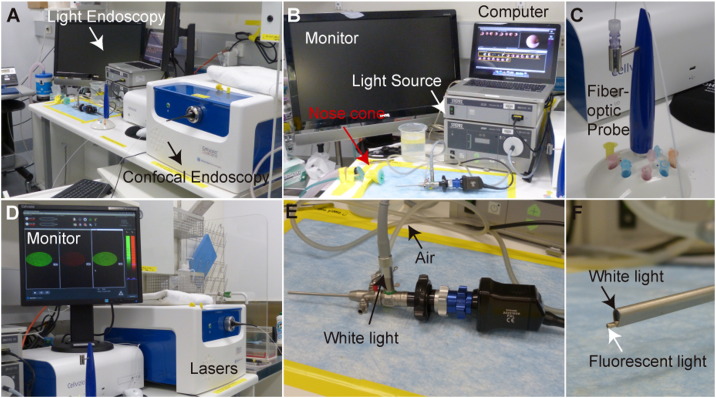
The white light and laser confocal microendoscopy systems. (A) The white light endoscopy system is run in parallel with the confocal endoscopy system. (B) The Karl Storz white light endoscopy system. A nose cone permits easy anesthesia administration. A monitor allows for easy visualization of the colon throughout the procedure. A light source permits visualization of the colon, and a standard computer is used to record videos of the procedure for subsequent analysis. (C) The CellVizio Mini-Z series fiber optic probe. (D) The CellVizio Dual Band endoscopy system. A monitor allows for easy visualization of the individual 488 and 660 laser channels, as well as the overlay, throughout the procedure. (E) The Karl Storz endoscopy unit, with a 3 mm endoscope sheath that allows for white light and air administration (for inflation) to the colon. (F) The combined use of the fiber optic confocal probe with the Karl Storz endoscopy sheath and white light.

**Fig. 2:**
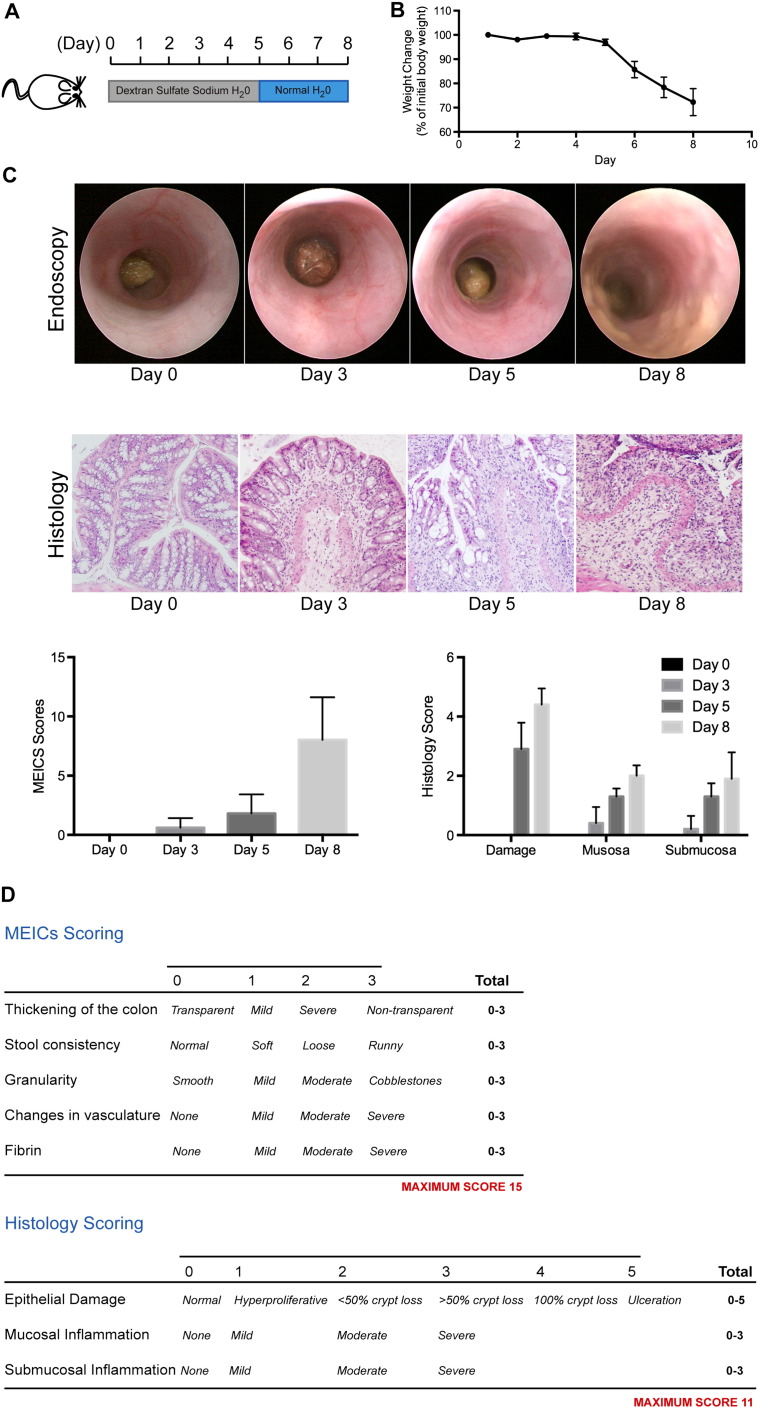
The DSS-induced acute colitis model. (A) Schematic representation of the 8 day dextran sulfate sodium (DSS)-induced mucosal damage and colitis protocol. (B) Progressive weight-loss is a clinical indication of disease severity (N = 5 wild-type mice). (C) Representative colonoscopy images obtained with the Karl Storz white light endoscopy system, and corresponding MEICS scores (N = 5 wild-type mice). Representative H&E images from the distal colon of mice euthanized at the indicated time-point, and corresponding histological scores (images taken with a 10 × objective; N = 5 wild-type mice). (D) A breakdown of the MEICS and histology scoring systems.

A clear workbench, ideally within a specific pathogen free animal facility, is required for the set-up of both of the endoscopy units (Fig. 1). This will permit routine monitoring of animals using both colonoscopy techniques with ease. The administration of DSS to mice ad libitum results in progressive weight-loss, and visible changes in the mucosal surface of the colon (Fig. 2A–C). These changes can be documented using the MEICS system (Becker et al., 2006), which provides a colonoscopy scoring parameter for changes in the thickness of the colon wall (transparency), stool consistency, vascularization, bleeding (indicated by fibrin) and regeneration (granularity; Fig. 2D). Importantly, the white light Karl Storz endoscopy system permits repeated monitoring of these parameters within a single mouse (Fig. 2C), providing an indication of the rate of progression of colitis.

In the absence of an available white light endoscopy system, changes in mucosal biology can be tracked through histopathology (Fig. 2C), and semi-quantified through a scoring system based on epithelial damage and inflammatory cell infiltrates visualized in H&E sections (Fig. 2D). As DSS-induced mucosal damage progresses, the incidence of ulceration and inflammation observed by histopathology increases, with maximal disease apparent on Day 8 of the protocol (Fig. 2C). In support of the use of endoscopy to monitor disease progression, the MEICS disease index generated through colonoscopy mirrors the disease score generated by histopathological observation of multiple cohorts of mice at defined time-points.

In contrast to the white light imaging achieved with the Karl Storz system, the CellVizio confocal microendoscopy system allows for more advanced imaging in real-time of fluorescent markers in live mice. The CellVizio laser scanning unit (class IIIB laser) generates excitation at both 488 nm and 660 nm, and couples the laser beam into a unique fiber optic probe chosen based on the application, with probe specs from 0.35 to 4.2 mm. Each fiber within the probe is a point detector, equivalent to a pinhole used in standard confocal imaging. In general, 9–50 frames per second are imaged up to a 1.4 μm lateral resolution, 10–70 μm optical section and 225–600 μm field of view, with a z-axis signal integration of 200 μm surface depth. This minimally invasive procedure can be applied, for example, to imaging of the brain, cornea, lung, heart, kidney, liver, pancreas, spleen and gastrointestinal tract (Vincent et al., 2006, Eser et al., 2011, Waldner et al., 2011, Everaert et al., 2012, Bruckner et al., 2014). Given the ease of access to the colon lumen by standard white light endoscopy, pairing of this technique with confocal endoscopy imaging of fluorescent markers can provide a unique view of the colon microenvironment (Becker et al., 2011, Schwitalla et al., 2013).

**Fig. 3:**
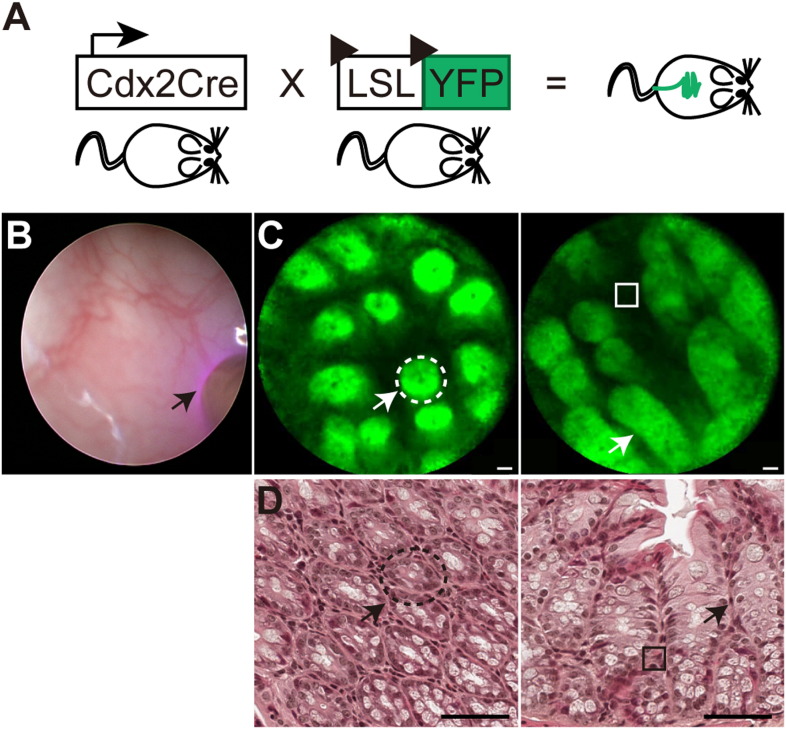
Confocal laser endoscopy tracking of fluorescently tagged epithelial cells. (A) Schematic representation of the constitutive CdxCre and floxed YFP alleles used in this study. (B) White light endoscopy (left) with the confocal probe placed against the mucosa and the laser initiated (black arrow). (C) Fluorescent epithelial cells and crypts (white arrows, dotted lines) and intraepithelial space (solid line). Scale bars = 20 μm. (D) Images of H&E sections of the tissue represented in (C). Epithelial cells and crypts (white arrows, dotted lines) and intraepithelial space (solid line). Scale bars = 50 μm.

Here we provide examples of imaging of the colonic mucosa and vasculature of mice. We have used mice heterozygote for *Cdx2*-Cre (Hinoi et al., 2007) and lox-stop-lox YFP (Srinivas et al., 2001), which have epithelial specific expression of YFP to image the colon using the 488 laser of the CellVizio unit (Fig. 3A). In order to guide entry into the colonic lumen of the mice initial videos of the colonic mucosa are captured using the Karl Storz white-light endoscopy system, this also permits appropriate placement of the confocal probe against the mucosal surface (Fig. 3B). Subsequent engagement of the 488 laser allows for visualization of the YFP-positive colonic epithelial cells (Fig. 3C; indicated arrow, Supplemental Video 1), including entire crypt structures and intra-epithelial spaces (Fig. 3C; indicated with open box). To orientate the reader, we have also provided the corresponding histological image of the epithelial crypt structures and intra-epithelial regions indicated (Fig. 3D).

**Fig. 4:**
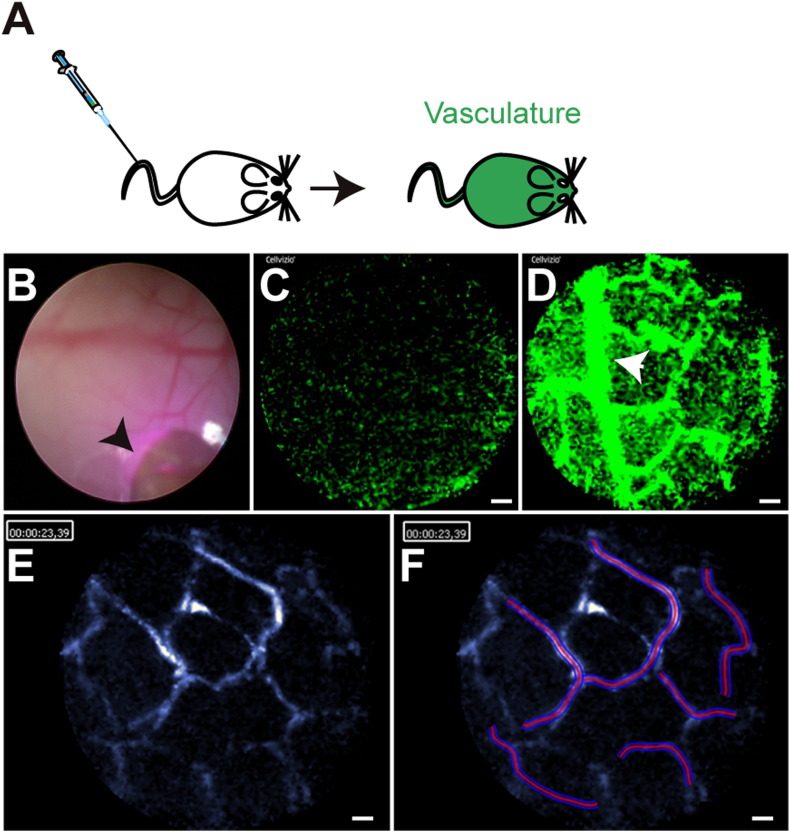
Confocal laser endoscopy tracking of fluorescently labeled vasculature. (A) Schematic representation of the administration of FITC–dextran to experimental mice. (B) White light endoscopy with the confocal probe placed adjacent to the distal colon mucosa (arrow). (C) Fluorescent image immediately after FITC–dextran administration. Scale bar = 20 μm. (D) Fluorescent vasculature approximately 10 min after FITC–dextran administration, with branching vessels indicated (arrow). Scale bar = 20 μm. (E) CellVizio vessel detection software conversion of image frame to blue scale in preparation for analysis. Scale bar = 20 μm. (F) CellVizio vessel detection software segmentation of vessels under 13 μm in diameter for analysis. Scale bar = 20 μm.

As an alternative to the use of transgenic animals with fluorescent reporters, fluorescently labeled antibodies or other reagents can be used to image the colonic mucosal by confocal microendoscopy (Becker et al., 2011, Schwitalla et al., 2013). Here we provide an example of FITC–dextran administered through intravenous tail vein injection, which permits the immediate visualization of the mucosal vasculature when imaged with the 488 laser using the CellVizio system (Fig. 4A). The use of the standard light endoscopy system permits the appropriate placement of the probe (Fig. 4B), and initiation of the laser allows for visualization of the movement of fluorescent dextran through the vasculature, which can be used to monitor vasculature formation and branching, blood flow and vasculature leakage (Fig. 4C–D; Supplemental Video 2). CellVizio has developed a user friendly “Vessel Detection” software package that permits segmentation analysis of microvessels, in addition to length, area and diameter measurements from the images collected (Fig. 4E–F). In the example provided, the average vessel diameter is 7.7 μm, with a total vessel area highlighted of 7, 330.97 μm^2^ (Fig. 4D).

## 5 Discussion

The use of pre-clinical animal models of IBD and CRC is essential to our understanding of the pathogenesis of the equivalent human diseases, and the discovery and validation of new tractable therapeutic targets. As the design of these animal models evolves to better recapitulate specific aspects of disease, our ability to interrogate the models has been enhanced by the development of new imaging techniques, including colonoscopy for mice.

Mouse colonoscopy provides a method to rapidly assess the progression of disease in an individual mouse. These methods are routinely used in mouse models of colitis and colon cancer (Putoczki et al., 2013). We have provided a standard DSS-induced protocol for the induction of mucosal damage, inflammation and colitis in mice, and demonstrated the consistency of the MEICS endoscopy scoring system with our standard epithelial damage and inflammation scoring system for H&E sections. While we do not advocate that colonoscopy is an alternative to histopathological analysis of disease, we do suggest that MEICS scores provide a suitable metric to monitor disease progression prior to ethical euthanasia of animals for complete histopathological analysis.

When standard white light endoscopy is coupled to confocal laser endoscopy, these techniques provide a means to observe microscopic architecture and specific cellular features within the colon in real-time. This provides numerous advantages: firstly, high resolution visualization of the mucosal layer will assist with the monitoring of non-neoplastic tissue and early identification of aberrant crypt foci, the precursor to polyp formation. Secondly, the kinetics of specific cell populations, for example mononuclear phagocytes recolonizing mucosa after depletion and adoptive transfer, can be monitored in real-time using reporter mice (Varol et al., 2009). Finally, and perhaps most importantly, transgenic reporter mice or fluorescently labeled molecules can be used to track the production of pathogenic factors like cytokines by specific cell populations, or to monitor the specificity and *in vivo* half life of new therapeutics within their target gastrointestinal cell population over time (Oh et al., 2014). Previously, imaging these processes in live mice was only possible through surface imaging, for example by two-photon microscopy, using large objective lenses. The CellVizio confocal endoscopy systems are also suitable for human clinical use (Atreya et al., 2014), meaning that the technical advancements made using mouse models can immediately be translated to patients.

Here we have provided a technical protocol to combine the minimally invasive standard white light endoscopy and laser scanning confocal endoscopy in mice, and have demonstrated the versatility of these techniques. The flexible CellVizio confocal probe can be used through the working channel of the Karl Storz system, within the limitation of the small field of view (~ 325 μm), and the scanning frame rate of 9 to 50 frames per second. However, CellVizio has developed software that permits stitching of images together to create a larger static image of a region of interest. Our results were generated using the Mini-Z series probe, which has a working distance of 50 μm and a lateral resolution of 3.5 μm to permit high resolution imaging of the epithelial cells and vasculature. However, a number of probes are available with a range of 1.4–3.5 μm lateral resolution, which would permit the visualization of individual immune cells. We have demonstrated the power of taking advantage of the 488 laser, and YFP or FITC–dextran monitoring; however, the CellVizio confocal systems are available with dual laser capabilities, which permit simultaneous monitoring of two parameters through use of both 488 and 660 nm excitation wavelengths.

## 6 Conclusion

The ability to perform high-resolution confocal imaging of the colon of live mice in situ over a defined time course will have a significant impact on biomedical research. By using the techniques described, our understanding of IBD and CRC onset and progression will be transformed through the generation of a new understanding of cell communication, trafficking and microenvironment relationships, in a manner not possible *ex vivo* or *in vitro*.

The following are the supplementary data related to this article.

Supplementary material.

Video 1 Confocal laser endoscopy tracking of fluorescently tagged epithelial cells.
Video recording of YFP positive epithelial cells and crypt structures in the distal colon from a mouse expressing YFP under the control of a constitutive CDX promoter. Scale bar = 20 μm.

Video 2 Confocal laser endoscopy tracking of fluorescently labeled vasculature.
Video recording of FITC dextran moving through the vasculature within the distal colon from a mouse intravenously injected approximately 10 min prior to imaging. Scale bar = 20 μm.

## Conflict of interest

The authors have none to declare.
