# Supplementary material for: Confocal laser endomicroscopy to monitor the colonic mucosa of mice
Source: J Immunol Methods. 2015 Jun;421:81–8. doi: 10.1016/j.jim.2015.04.012 (PMC5803490; doi:10.1016/j.jim.2015.04.012)
Supplement: Supplementary file 1 — Supplementary material. [file mmc1.pdf]

Confocal laser endomicroscopy to monitor the colonic mucosa of mice

Lisa Mielke[1,2], Adele Preaudet[1], Gabrielle Belz[1,2], Tracy Putoczki[1,2,*]

[1]The Walter and Eliza Hall Institute for Medical Research, Melbourne, Australia

[2]The Department of Medical Biology, University of Melbourne, Australia

INVENTORY of SUPPLEMENTAL INFORMATION

Supplemental Figure 1

Supplemental Video 1

Supplemental Video 2

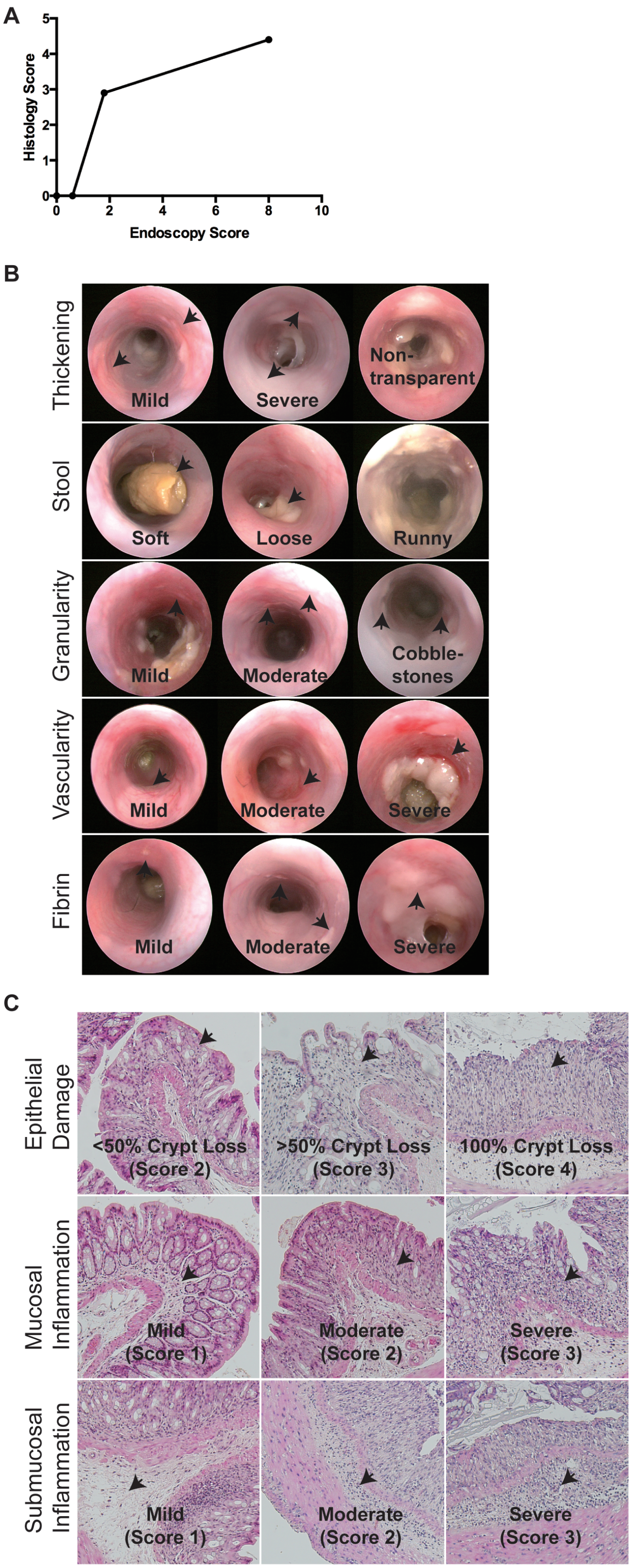

Supplemental Figure 1.

(A) Correlation between the histology scores and the endoscopy scores presented in Figure 1.

(B) Example endoscopy images and scores from mice with different colitis severities.

(C) Example histology images and scores from mice with different colitis severities. (Images were taken with a 10X objective)

SUPPLEMENTAL VIDEOS

<insert Supplemental Video 1>

Video 1. ***Confocal laser endoscopy tracking of fluorescently tagged epithelial cells.***
Video recording of YFP positive epithelial cells and crypt structures in the distal colon from a mouse expressing YFP under the control of a constitutive CDX promoter. Scale bar=20 μm.

<insert Supplemental Video 2>

Video 2. ***Confocal laser endoscopy tracking of fluorescently labeled vasculature.***
Video recording of FITC dextran moving through the vasculature within the distal colon from a mouse intravenously injected approximately 10 minutes prior to imaging. Scale bar=20 μm.
